# Reassessing Interkingdom Horizontal Gene Transfer Suggests Limited Influence on Plant Genomes

**DOI:** 10.1002/ece3.72653

**Published:** 2025-12-17

**Authors:** Kevin Aguirre‐Carvajal, Vinicio Armijos‐Jaramillo

**Affiliations:** ^1^ Bio‐Cheminformatics Research Group Universidad de Las Américas Quito Ecuador; ^2^ Computer Science Faculty, University of A Coruna, CITIC‐Research Center of Information and Communication Technologies A Coruña Spain; ^3^ Carrera de Ingeniería en Biotecnología, Facultad de Ingeniería y Ciencias Aplicadas Universidad de Las Américas Quito Ecuador

**Keywords:** anomalous phylogenies, interkingdom horizontal gene transfer, plants, reassess

## Abstract

Horizontal gene transfer (HGT) is a well‐established mechanism of genetic innovation in bacteria, but its impact on eukaryotes—and particularly on plants—remains debated. In recent years, numerous studies have reported hundreds of putative nuclear genes in plants with origins in other kingdoms, often interpreted as adaptive acquisitions. Most of these claims rely on phylogenetic reconstructions, which are highly sensitive to taxon sampling and can shift as new homologs are identified. To reassess this evidence, we systematically collected published reports of interkingdom HGT in plants and reconstructed phylogenetic trees using up‐to‐date genomic data from public databases. Candidate topologies were first evaluated with an automated tool and then manually curated. Our reanalysis shows that only 29.3% of previously reported cases remain consistent with an interkingdom HGT scenario. Many candidates are more parsimoniously explained by alternative processes such as gene loss or incomplete taxon sampling. These findings highlight the dynamic nature of phylogenetic inference and caution against treating HGT as the default explanation for anomalous phylogenies in plant genomes.

## Graphical abstract

Horizontal gene transfer (HGT) has been proposed as an important contributor to plant genome evolution, with hundreds of candidate genes reported across kingdoms. By reanalyzing published cases with updated genomic data and phylogenetic reconstructions, we found that only 29.3% remain consistent with interkingdom HGT. Many reported cases are better explained by alternative processes, underscoring the need for caution in interpreting anomalous phylogenies as evidence of HGT.

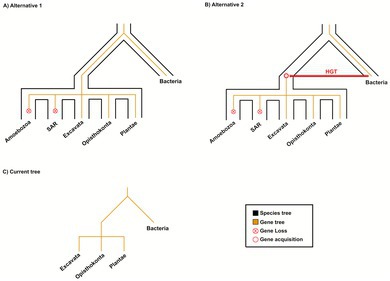

## 1 Introduction

Horizontal gene transfer (HGT)—the acquisition of genetic material from unrelated species without reproduction mechanisms involved—has long been recognized as a major driver of adaptation in bacteria (Dmitrijeva et al. 2024). In contrast, HGT in multicellular eukaryotes was traditionally considered anecdotal, especially when involving transfers across kingdom boundaries. However, with advances in sequencing technologies and the increasing availability of genomic data, interkingdom HGT has been widely reported in various eukaryotic lineages, including Plantae (Yue et al. 2012; Ma et al. 2022; Haimlich et al. 2024; Wu et al. 2024). The likelihood of interkingdom HGT has been hypothesized to increase in contexts involving close biological interactions, such as symbiosis, commensalism, or endosymbiosis. However, there is currently no solid evidence that long‐term lateral transfers are stably retained through these relationships (Keeling 2024). Moreover, the molecular mechanisms underlying the transfer of genetic material between distantly related species remain largely enigmatic. Several potential mediators have been proposed—including viruses (Malik et al. 2017), transposable elements (Urquhart et al. 2023), extracellular membrane vesicles (Douanne et al. 2022; Marcilla and Sánchez‐López 2022), and tunneling nanotubes (Haimovich et al. 2021)—yet none have been directly observed facilitating genetic exchange across kingdom boundaries. Recently, Urquhart et al. (2025) demonstrated that *Starship* transposable elements can mediate horizontal transfer between unrelated fungal species under natural conditions. Nevertheless, neither this nor any comparable mechanism has been shown to operate between kingdoms. To date, the only well‐documented example of natural interkingdom HGT is the transfer of genetic material from *Agrobacterium* spp. to plants, which occurs via a plasmid‐mediated system rather than any of the previously proposed vectors.

The reported prevalence of interkingdom HGT in plants is surprising, given that plant biology presents several inherent barriers to the acquisition and stable maintenance of foreign DNA. Features such as rigid cell walls, compartmentalized genomes, and tightly regulated cellular environments significantly reduce the likelihood of successful gene transfer and integration. Moreover, for a horizontally acquired gene to persist, it must not only integrate into the host genome but also be properly expressed, translated, and, in many cases, correctly folded and targeted to its functional location within the cell. A well‐established exception to these constraints is endosymbiotic gene transfer (EGT)—the movement of genes from organelles of bacterial origin, such as mitochondria and chloroplasts, to the nuclear genome (Katz 2015; Ku et al. 2015). Despite these theoretical and mechanistic limitations, an increasing number of studies have proposed that HGT from free‐living organisms may have contributed adaptively to plant genome evolution (Yang et al. 2015; Wickell and Li 2020; Ma et al. 2022).

For instance, Yue et al. (2012) proposed that at least 57 nuclear gene families were horizontally transferred from various organisms to the ancestors of land plants. These genes are thought to have played a critical role in the transition from aquatic to terrestrial environments, as they encode enzymes involved in key biological functions such as starch biosynthesis, polyamine and hormone metabolism, xylem formation, and plant defense mechanisms.

A classical and well‐documented example of interkingdom HGT is the transfer mediated by *Agrobacterium tumefaciens* and *A. rhizogenes* , whose tumor‐inducing (Ti) and root‐inducing (Ri) plasmids, respectively, integrate foreign DNA into the host plant genome (Chilton et al. 1977, 1982). Another frequently cited case is the TAL‐type transaldolase (TAL) gene, implicated in the pentose phosphate pathway, which Yang et al. (2015) proposed to be of bacterial origin. More recently, Haimlich et al. (2024) identified at least 59 genes of apparent bacterial origin across various plant species. However, the authors themselves expressed caution regarding these findings, acknowledging the methodological limitations of phylogenetic approaches in accurately detecting interkingdom HGT events.

Such caution is inherent to the phylogenetic methods commonly employed to identify HGT candidates. Beyond the general limitations associated with phylogenetic reconstruction, the patchy taxonomic distributions often observed in putative HGT trees may also be explained by gene loss events or undetected paralogy (Yue et al. 2012; Haimlich et al. 2024). More recently, Aguirre‐Carvajal et al. (2024) emphasized that the absence of homologs at the time of analysis can further obscure accurate HGT detection in fungi. Additionally, contamination in genomic datasets and annotation errors can significantly increase the likelihood of misidentifying genes as horizontally transferred.

Given the ongoing debate surrounding the significance of horizontal gene transfer—particularly interkingdom HGT—it is crucial to pause and critically reassess the existing evidence in Plantae. In this study, we revisited previously reported interkingdom HGT candidates in plants and reanalyzed them in light of the expanded genomic data now available. Our aim was to evaluate whether the growth of genomic databases alters the interpretation of HGT events and to assess whether interkingdom HGT truly plays as substantial a role in plant evolution as some studies have suggested.

## 2 Methods

To assess the significance of interkingdom horizontal gene transfer (HGT) in Plantae, we conducted a comprehensive literature survey encompassing all relevant publications available to date. For this purpose, we performed an advanced search in the PubMed database, using the search strategies detailed in Table 1, with a cutoff date of April 2025.

**TABLE 1:** Results of PubMed searches and number of articles retrieved.

Search	Number of articles
(“viridiplantae”[Title/Abstract] OR “vascular plants”[Title/Abstract] OR “nonvascular plants”[Title/Abstract] OR “land plants”[Title/Abstract] OR “seed plants”[Title/Abstract] OR “green algae”[Title/Abstract]) AND “lateral gene transfer”[Title/Abstract]	23
(“viridiplantae”[Title/Abstract] OR “vascular plants”[Title/Abstract] OR “nonvascular plants”[Title/Abstract] OR “land plants”[Title/Abstract] OR “seed plants”[Title/Abstract] OR “green algae”[Title/Abstract]) AND “horizontal gene transfer”[Title/Abstract]	137
(“Viridiplantae”[Title/Abstract] OR “land plants”[Title/Abstract] OR “green algae”[Title/Abstract]) AND “horizontal gene transfer”[Title/Abstract] AND “Interdomain”[Title/Abstract]	1
(“green algae”[Title/Abstract] OR “land plants”[Title/Abstract] OR “viridiplantae”[Title/Abstract]) AND “lateral gene transfer”[Title/Abstract]	23
(“land plants”[Title/Abstract] OR “viridiplantae”[Title/Abstract] OR “green algae”[Title/Abstract]) AND “horizontal gene transfer”[Title/Abstract]	120
“Viridiplantae”[Title/Abstract] AND “horizontal gene transfer”[Title/Abstract]	20
“Viridiplantae”[Title/Abstract] AND (“horizontal gene transfer”[Title/Abstract] OR “lateral gene transfer”[Title/Abstract] OR “HGT”[Title/Abstract])	21
(“horizontally acquired genes”[Title/Abstract] OR “lateral gene transfer”[Title/Abstract]) AND “plants”[Title/Abstract]	121
(“horizontal gene transfer”[Title/Abstract] OR “lateral gene transfer”[Title/Abstract]) AND “plants”[Title/Abstract] AND “gene acquisition”[Title/Abstract]	5
(“horizontal gene transfer”[Title/Abstract] OR “lateral gene transfer”[Title/Abstract]) AND “plants”[Title/Abstract] AND “microorganisms”[Title/Abstract]	76
(“horizontal gene transfer”[Title/Abstract] OR “lateral gene transfer”[Title/Abstract]) AND “plants”[Title/Abstract] AND “phylogeny”[Title/Abstract]	81
(((“horizontal gene transfer”[Title/Abstract] OR “lateral gene transfer”[Title/Abstract]) AND “plants”[Title/Abstract] AND “interdomain”[Title/Abstract]) OR “interkingdom”[Title/Abstract]) AND “Novel”[Title/Abstract]	156

All search results were obtained using easyPubMed v2.13 (Fantini 2023). After removing duplicate entries based on their PubMed identifiers (PMIDs), a total of 542 unique articles were retained (see Table S1). We then applied strict inclusion and exclusion criteria, selecting only studies that: (i) primarily focused on horizontal gene transfer (HGT), (ii) reported interkingdom HGT events involving Plantae as the recipient lineage, (iii) did not duplicate candidate genes across publications, and (iv) provided data compliant with FAIR principles (Findable, Accessible, Interoperable, and Reusable). Following this curation, 35 articles were selected, from which a total of 1170 HGT candidates were extracted. A complete bibliographic summary is available in Table S1.

For each HGT candidate, homology searches were conducted using DIAMOND BLASTp v2.1.9, following the parameters recommended by AVP v1.0.10 (Koutsovoulos et al. 2022): ‐k 500 ‐‐evalue 1e‐5 ‐‐outfmt 6 qseqid sseqid pident length mismatch gapopen qstart qend sstart send evalue bitscore staxids. All homology searches were conducted using the NCBI NR database in combination with Phytozome (Goodstein et al. 2012), and the plant proteomes deposited in Genome Warehouse (Ma et al. 2025). Each of these databases was updated to its September 30th, 2025 release.

In addition, we employed AVP v1.0.10 to assess whether the recovered candidates were identified as HGT cases using an automated, phylogeny‐based method. The Ingroup parameter for this analysis was set to “Viridiplantae” representing the lineage where the potential gene transfer occurred. The exclusion group parameter (EGP) was defined based on the HGT recipient lineage reported in the original studies. The specific parameters used for each candidate are listed in Table S2. The EGP defines the taxonomic groups excluded from the identification of relevant homologs for detecting HGT events.

The candidates were analyzed using AVP in IQ‐TREE mode to systematically evaluate the phylogenetic topologies generated for each case. Based on these topologies, the tool categorized each candidate as HGT, NoHGT, or Complex, depending on the phylogenetic patterns observed.

Additionally, for each candidate, we reused the homolog search results generated by AVP, retaining only those sequences that met the following thresholds: pairwise similarity above 30%, query coverage exceeding 60%, and an *e* value lower than 1 × 10^−5^. The filtered homologous sequences were retrieved using NCBI EFETCH v16.2. Multiple sequence alignments were then performed with MAFFT v7.520 (Katoh and Standley 2013) in “Auto” mode, and subsequently refined using Gblocks v0.91b (Talavera and Castresana 2007) with default settings. Alignments were converted to Phylip format for downstream analyses. Model selection was conducted with modeltest‐ng v0.1.7 (Darriba et al. 2020) to identify the best‐fitting substitution model for each dataset. Phylogenetic trees were then inferred using PHYML v3.3 (Guindon et al. 2010), including Shimodaira–Hasegawa‐like branch support, and outputting results in Newick format.

Following the reconstruction of phylogenetic trees for each candidate, taxonomic information for the homologous sequences was retrieved from NCBI using custom scripts developed in Python 3.10.12. This information was used to generate phylogenetic trees in Nexus format, which were manually inspected in Geneious Prime 2023.2.1 (https://www.geneious.com [18 July 2025]) to assess potential cases of horizontal gene transfer.

Finally, a contamination assessment was carried out for candidates lacking homologs in other eukaryotes. This involved examining the homologs of genes flanking each candidate, as well as the length of the contigs on which they were located. BLASTp searches (v2.13.0, accessed August 1, 2025; https://blast.ncbi.nlm.nih.gov/) were performed on the translated upstream and downstream genes to evaluate whether the surrounding genomic context was consistent with that of the proposed donor lineage. When the flanking genes also corresponded to the putative donor lineage, the candidate was classified as potential contamination; in contrast, if this was not the case, it was retained as a likely genuine HGT candidate. To evaluate the likelihood that a candidate originated from an assembly issue, we collected assembly metrics for each genome/candidate when such information was available in public databases. All retrieved statistics are presented in Table S3.

## 3 Results

From the 35 articles reporting interkingdom HGT, we compiled 1170 candidate genes proposed to have been transferred to plant species. Reanalysis with the AVP software classified 433 candidates as showing an HGT signal, 18 as Complex, and 718 as NoHGT. This group comprises one candidate that could not be assessed due to the absence of its sequence in the original publication, along with 5 sequences that lacked sufficient homologs to enable phylogenetic reconstruction. Detailed AVP results are provided in Table S4.

**FIGURE 1:**
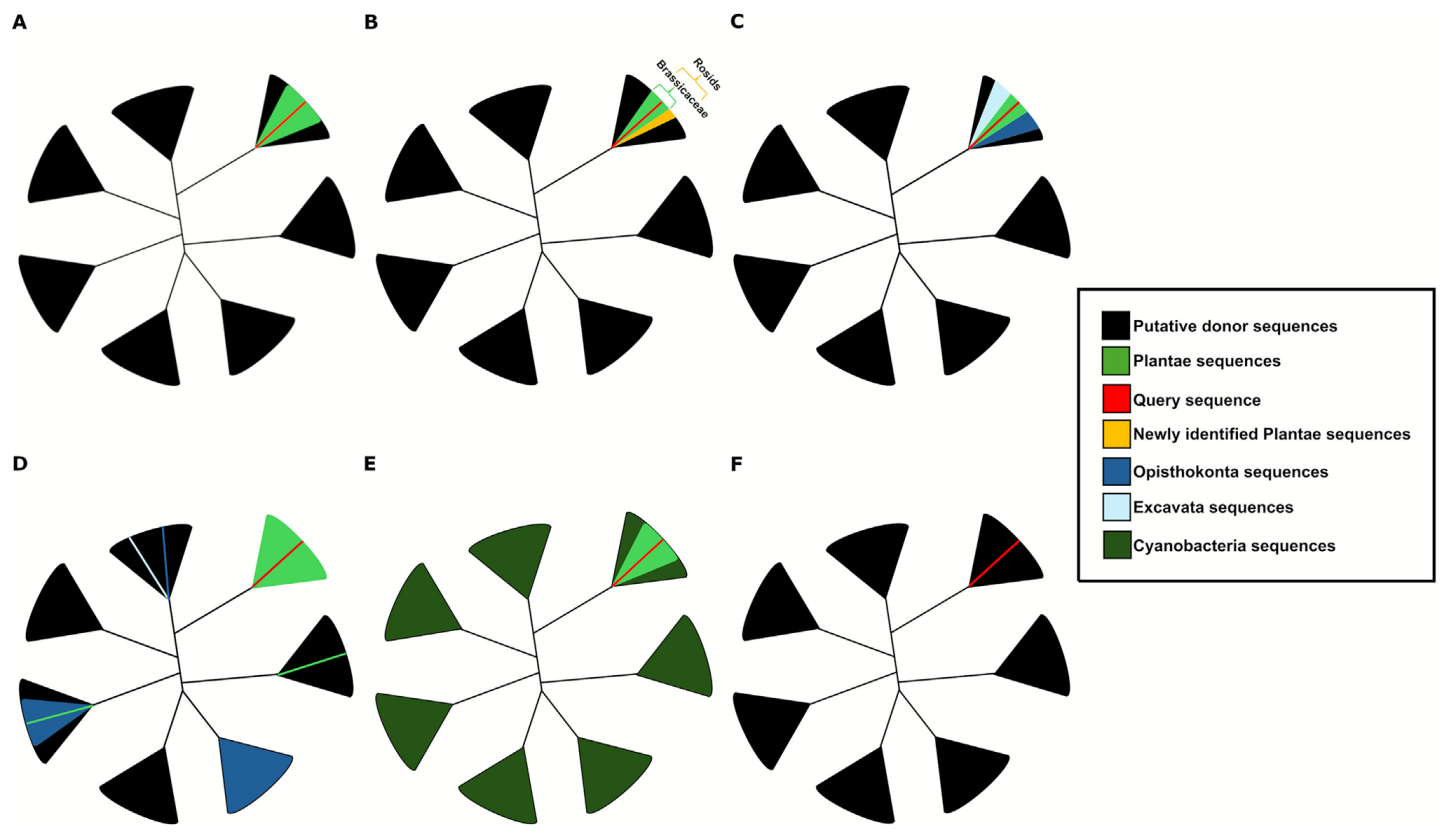
Representative phylogenetic patterns observed during the reanalysis of interkingdom HGT candidates in plants. (A) HGT: Plant sequences, including the query, form a clade surrounded by sequences from a different kingdom, with no additional eukaryotic homologs detected. (B) HGT_recipient_shifted: Similar to (A), but new homologs are identified in other plant lineages, shifting the putative timing or lineage of transfer (e.g., from Brassicaceae to the broader Rosid clade). (C) NoHGT: Candidate sequences group in a monophyletic group with homologs from multiple eukaryotic kingdoms. Explaining this as interdomain HGT would require ancient transfer at, or near, the eukaryotic ancestor followed by widespread gene loss; alternatively, vertical inheritance with differential gene loss provides a simpler explanation. (D) Inconclusive: Patchy or polyphyletic distributions across several kingdoms, preventing robust interpretation. (E) Putative endosymbiotic gene transfer (EGT): Candidates cluster only with plant and cyanobacterial sequences, consistent with plastid‐to‐nuclear transfer. (F) Potential contamination: Only one candidate appears in plants and show high similarity to prokaryotic or nonplant sequences; further analyses of genomic context were used to confirm classification. All patterns are schematically represented as collapsed, unrooted trees, based on data available in https://zenodo.org/records/17552462 and detailed in Table S4.

All results were manually reviewed, candidate by candidate, to validate or adjust the AVP classifications. During this reassessment, we identified recurring patterns, which we grouped into the following categories: HGT, HGT_recipient_shifted, inconclusive, potential contamination, putative_EGT, and NoHGT. A detailed description of each category is provided below, and a schematic overview is shown in Figure 1.

- The HGT category included candidates for which no additional homologs were found outside the originally reported recipient clade, and whose phylogenetic topology remained consistent with an interkingdom HGT scenario (Figure 1A).
- The HGT_recipient_shifted category referred to candidates for which new Plantae homologs, beyond those originally reported, were identified. These sequences formed a monophyletic group with the candidate, indicating that the putative transfer may have occurred earlier in evolutionary history than initially proposed (Figure 1B).
- The NoHGT category was assigned to candidates whose homologs were identified in other major eukaryotic clades (e.g., Excavata, Opisthokonta, Amoebozoa, SAR [Stramenopila, Alveolata, and Rhizaria], or others), yielding a pattern more consistent with vertical inheritance. We consider phylogenies containing homologs from more than one eukaryotic kingdom difficult to explain as interdomain HGT (i.e., transfers from prokaryotes), and those with homologs from more than two kingdoms even less so. In such cases, the transfer would have to occur in, or near, the last common ancestor of eukaryotes, followed by multiple independent gene loss events across the remaining kingdoms. Such topologies can often be explained without invoking HGT, relying solely on differential gene loss as the cause (Figure 1C).
- The Inconclusive category comprised cases in which the phylogenetic tree topology did not allow a definitive interpretation. Most candidates in this group displayed patchy distributions that could be explained by multiple scenarios. This category also includes instances where no tree could be constructed due to the absence of detectable homologs (Figure 1D).
- The Putative EGT (endosymbiotic gene transfer) category was assigned to cases where the query sequence clustered exclusively with cyanobacterial homologs, indicating a likely plastid‐derived endosymbiotic transfer (Figure 1E).
- The Potential Contamination category included candidates lacking detectable homologs in eukaryotes, showing high similarity to prokaryotic sequences, and/or located on short contigs flanked upstream and downstream by genes clearly originating from a kingdom other than Plantae (Figure 1F).

Of the 1170 candidates analyzed, 19.66% (230) were classified as HGT, 9.66% (113) as HGT_recipient_shifted, 30.6% (358) as Inconclusive, 1.11% (13) as putative EGT, 36.84% (431) as NoHGT, and 2.14% (25) as Potential Contamination (see Table S4 for detailed phylogenetic analysis). Our classifications matched the AVP results in 80% of noninconclusive cases; however, manual curation allowed us to recover and interpret tree topologies under a wider range of evolutionary scenarios that the automated tool could not detect. All multiple sequence alignments and reconstructed phylogenetic trees generated in this study are accessible in https://zenodo.org/records/17552462, and the classification of the observed phylogenetic patterns is presented in Table S4.

Additionally, we categorized the HGT candidates according to major *Plantae* lineages and included, for each group, the total number of described species and available complete genomes as reported by NCBI (Table S5). No significant correlation was found between the number of validated HGT candidates (HGT + HGT_recipient_shifted) or refuted cases (NoHGT + potential contamination) and the number of genomes available per lineage. Nonetheless, we observed a relatively higher proportion of refuted HGT cases in Hornworts, Chlorophyta, and Gymnosperms, whereas Angiosperms and Liverworts showed a greater proportion of validated candidates. As this study integrates data from multiple sources with differing sampling strategies and analytical goals, these lineage‐specific patterns should be interpreted cautiously, as they may reflect underlying data or sampling biases rather than true biological trends.

## 4 Discussion

In this study, we reanalyzed all reported interkingdom HGT candidates in Plantae species for which data were available. Our objective was to assess the number of HGT from distantly related organisms on current plant genomes. Although 1170 candidate genes have been reported in the literature, our updated analyses detected phylogenetic signals consistent with interkingdom HGT in only 343 cases (29.3%). This trend closely mirrors findings from a recent reanalysis of the fungal subphylum Pezizomycotina (Aguirre‐Carvajal et al. 2025).

Phylogenetic reconstruction is often regarded as the gold standard for detecting HGT (Chan et al. 2017), particularly when transfers are assumed to have become fixed long ago and are indistinguishable from vertically inherited genes based on genomic features (Keeling and Palmer 2008). However, the reliability of this approach depends heavily on the availability and completeness of homologous sequences. Our reanalysis shows that many interkingdom HGT cases once considered robust become unlikely when reassessed with updated genomic data. This highlights not only the limitations of phylogenetic methods in the absence of comprehensive databases but also the risk of systematically overestimating the role of HGT in plant evolution.

Our reanalysis revealed several noteworthy patterns in the phylogenetic tree topologies. The first, and most straightforward, was the topology consistent with interkingdom HGT. These candidates exhibited remarkable stability over time: despite the inclusion of additional homologs, their tree structures remained congruent with expectations for HGT. Nevertheless, this stability may not be permanent, as future database expansions could alter the interpretation of these cases. For now, we regard these candidates as consistent with interkingdom HGT, while emphasizing the importance of continuous reassessment to clarify their true evolutionary origins.

We observed a recurrent pattern that does not dismiss the interkingdom HGT hypothesis but rather modifies the inferred antiquity and placement of the putative transfer in the phylogenetic gene tree. We designate this as the “HGT_recipient_shifted” pattern. In such cases, additional homologs were recovered exclusively within plants, but absent from other eukaryotic lineages, thereby shifting the phylogenetic context in which the transfer is interpreted. A representative example is candidate AEE29437.1, originally reported by Haimlich et al. (2024) as a transfer restricted to Brassicales. With the addition of newly available homologs in Chlorophyta, the most parsimonious interpretation is that the transfer, if it occurred, must have taken place much earlier in plant evolution, near the base of the Plantae lineage. Importantly, the interdomain HGT scenario remains consistent here, since the expanded set of plant sequences forms a monophyletic group with the original candidates, embedded among bacterial homologs. This pattern underscores how the continuous expansion of genomic databases can refine—not necessarily overturn—initial HGT hypotheses, by providing a more accurate placement of the evolutionary event within the plant phylogeny.

**FIGURE 2:**
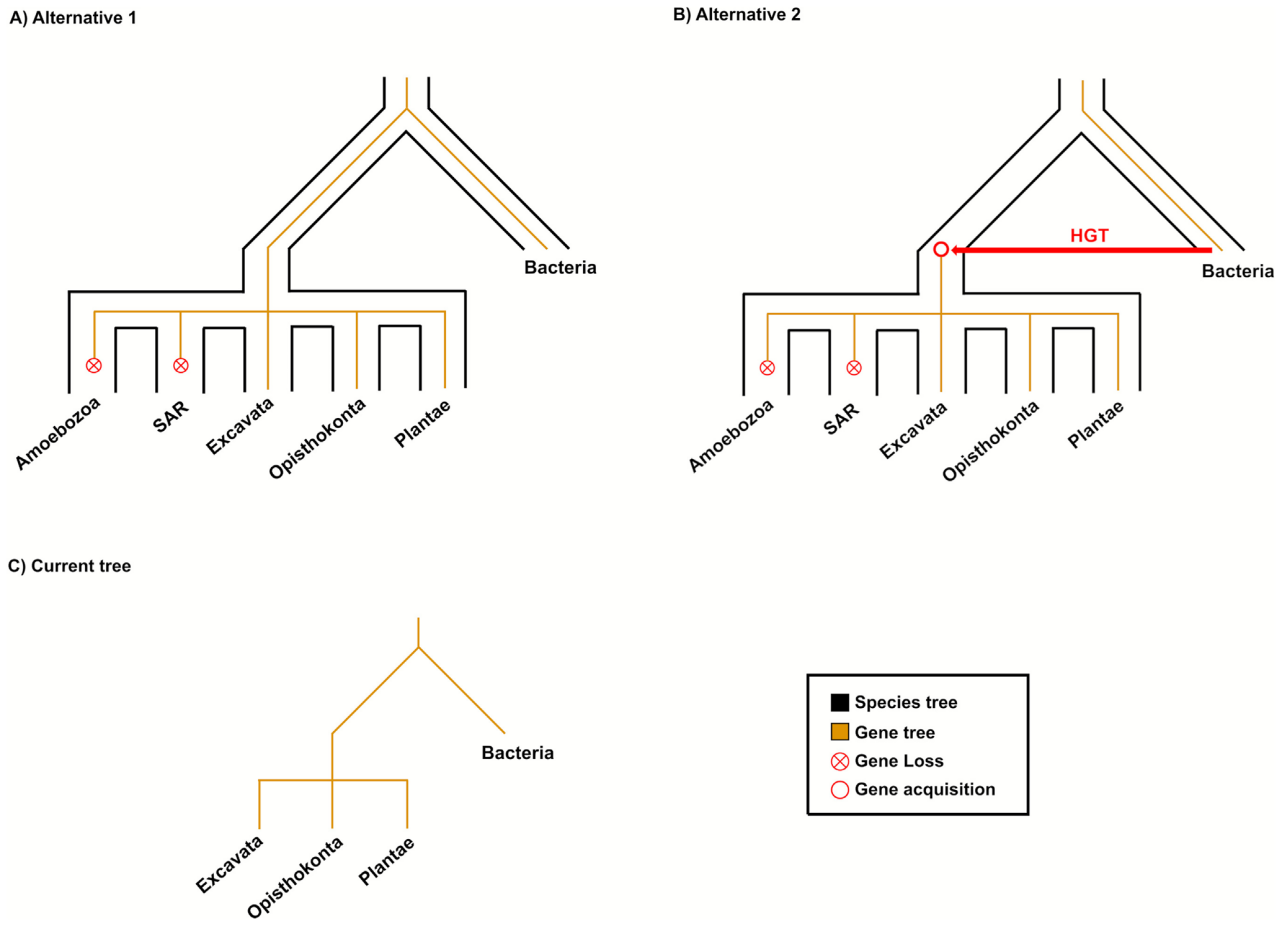
Hypothetical examples illustrating two possible explanations for the NoHGT pattern described in this study. (A) Alternative 1: The gene was present in both prokaryotes and eukaryotes and transmitted vertically, but subsequently lost in the SAR (Stramenopila, Alveolata, and Rhizaria) and Amoebozoa lineages. (B) Alternative 2: The gene was acquired by horizontal transfer from bacteria into an ancestral eukaryote, retained in Plantae, Opisthokonta, and Excavata, but lost in SAR and Amoebozoa. (C) Current tree: The resulting phylogeny appears identical, underscoring the difficulty of distinguishing vertical inheritance with gene loss from ancient HGT.

In contrast, we also identified a recurrent pattern that no longer supports the interkingdom HGT hypothesis. Within this category, several distinct scenarios emerge. One of the most frequent involves the recovery of new homologs for the proposed HGT candidate, which fundamentally reshapes the phylogenetic signal. For instance, in the case of *Tawa019950* from Wu et al. (2024), the addition of new sequences reconstructs a monophyletic group spanning multiple eukaryotic lineages. This topology shifts the apparent origin of the gene to the root, or close to the root, of eukaryotes. Under this interpretation, HGT becomes a far less parsimonious explanation, as the observed distribution can be more simply explained by vertical inheritance from an ancestral eukaryotic gene followed by multiple independent gene losses across major lineages. The HGT hypothesis, by contrast, would require invoking an ancient transfer from bacteria into a eukaryotic ancestor, also followed by widespread losses—an even less plausible scenario (Figure 2).

A related situation occurs when homologs are identified in eukaryotic kingdoms other than the originally proposed recipient and the putative donor. In such cases, the HGT hypothesis would imply that the same gene was transferred independently into two or more eukaryotic kingdoms, as illustrated by the *Sphmag17G037800* candidate (Wu et al. 2024). A more coherent explanation, however, is that the gene was already present in ancestral eukaryotes, but subsequently underwent dramatic lineage‐specific expansions in some groups—those initially interpreted as donors—while remaining rare or patchily distributed in others, such as Plantae. This scenario highlights how expanding genomic sampling can transform a signal once interpreted as interdomain HGT into a more consistent pattern of vertical inheritance with lineage‐specific retention or loss.

The group of candidates we classified as “potential contamination” includes sequences for which no additional homologs could be retrieved from eukaryotes, and for which the original authors were also unable to provide supporting evidence. We also placed into this category those candidates located on short contigs, flanked by neighboring genes clearly assigned to kingdoms other than Plantae. In such cases, contamination during sequencing or assembly becomes a far more plausible explanation than genuine interkingdom transfer. Indeed, contamination from exogenous organisms is a well‐documented issue in genome projects (Merchant et al. 2014) and, if not carefully accounted for, can artificially inflate the apparent number of HGT events. For this reason, we take a conservative stance and prefer to treat such cases with caution rather than risk perpetuating spurious claims of horizontal transfer.

Finally, we identified 13 candidates that exhibited signatures consistent with endosymbiotic gene transfer (EGT)—that is, the movement of genes from plastids or other organelles into the nuclear genome of plants. These candidates showed clear similarity to cyanobacterial sequences, but no detectable homology to genes from noncyanobacterial bacteria or from eukaryotic lineages outside Plantae. While the possibility of a direct horizontal transfer from cyanobacteria cannot be entirely ruled out, the well‐established cyanobacterial origin of plastids (Keeling 2009) and their intimate physical association with the host nuclear genome make an EGT explanation more parsimonious. Supporting this view, we also verified that all of these candidates are located on contigs confidently assigned to nuclear genomes, rather than to chloroplast assemblies. Taken together, these cases highlight the need to clearly distinguish EGT from genuine interkingdom HGT, in order to avoid overestimating the extent of bacterial‐to‐plant gene transfer.

Interestingly, we found 776 reported candidates of interkingdom transfer from prokaryotes, 280 from fungi, 60 from Metazoa, but only 28 from viruses. These numbers suggest that bacteria and fungi are apparently more common sources of transferred genes to plants than viruses. This is striking, given that viruses are uniquely equipped to integrate their genetic material directly into host genomes, whereas only a few bacteria (e.g., *Agrobacterium tumefaciens* ) can achieve this—and only in specific hosts. How, then, could bacteria and fungi account for so many more cases of HGT than viruses? One possibility is that past evolutionary dynamics created conditions that favored these transfers. Alternatively, what we are observing may not be true interkingdom HGT events, but instead phylogenetic artifacts that generate HGT‐like topologies. Our reanalysis supports the latter view: in many cases, the inclusion of newly available homologs reshapes phylogenetic trees, undermining earlier HGT hypotheses and urging a reconsideration of how we interpret anomalous tree reconstructions.

Despite the inherent uncertainty introduced by the incomplete representation of homologs in current databases, there remains a pressing need for analytical approaches capable of distinguishing among alternative evolutionary scenarios inferred from gene trees. One widely explored strategy is gene–species tree reconciliation, which can, in principle, differentiate between HGT and gene duplication or loss. However, its empirical application—for instance, in fungi—has demonstrated limited statistical power for detecting HGT (Dupont and Cox 2017). Moreover, such algorithms often fail to adequately account for incomplete lineage sorting (ILS), a common source of phylogenetic incongruence. To address this limitation, more sophisticated frameworks have been proposed that reconcile locus networks, species networks, and gene trees under a coalescent model, allowing for the joint inference of ILS, gene duplication/loss, and hybridization (Du et al. 2019). While promising, these approaches are computationally intensive and remain impractical for large‐scale genomic datasets, such as those analyzed in this reanalysis. Consequently, complex evolutionary scenarios—such as gene loss followed by reacquisition through HGT, or HGT events arising from secondary or tertiary endosymbiotic episodes—must, for now, be inferred primarily from single‐gene phylogenies, with full acknowledgment of the methodological and interpretative uncertainties inherent to this approach.

Several studies have argued that reports of interkingdom HGT in the scientific literature are frequently overestimated (Ku and Martin 2016; Martin 2017; Aguirre‐Carvajal et al. 2024, 2025), and our findings point in the same direction. This does not imply that interkingdom HGT does not occur; rather, it highlights the need for caution. When anomalous phylogenies are recovered, HGT should be considered as one possible explanation, but not the first or default one. The central contribution of this study is to emphasize that alternative hypotheses—such as gene loss, incomplete lineage sorting, or database limitations—must be carefully explored before invoking interkingdom transfers of genetic material.

It is well established that phylogenetic reconstructions carry inherent limitations (Som 2015), and therefore conclusions drawn from these analyses must be approached with caution. In the case of ancient interkingdom HGT, we are essentially trying to infer hundreds of millions of years of evolutionary history from a single‐gene family. Over such vast timescales, the erosion of phylogenetic signal is inevitable, making robust inference challenging. Consequently, alternative explanations—such as ancestral gene loss or incomplete lineage sorting—must be carefully considered before attributing patterns solely to HGT. In our reanalysis, we therefore classified a substantial fraction of candidates as “inconclusive,” not as a dismissal of HGT, but as a recognition that the data simply do not support strong evolutionary claims at this stage.

## 5 Conclusion

In this study, we reanalyzed a broad set of published reports describing interkingdom HGT events in Plantae, with the aim of reassessing the evolutionary impact of this phenomenon. Using a phylogenetic approach, we found that only about 29% of the reported candidates remain consistent with an interkingdom HGT scenario. Even so, we emphasize that these candidates should be periodically reexamined as genomic databases continue to expand. A substantial fraction of cases displayed patchy phylogenetic distributions, suggesting complex evolutionary histories that may or may not involve HGT, while the remainder were incongruent with an interkingdom transfer hypothesis. Taken together, our findings underscore the need to critically reassess the role of interkingdom HGT in plant evolution and to reevaluate reported candidates in light of new genomic data as it becomes available.

## Author Contributions

**Kevin Aguirre‐Carvajal:** data curation (lead), formal analysis (equal), investigation (equal), methodology (lead), resources (lead), software (lead), writing – original draft (supporting), writing – review and editing (supporting). **Vinicio Armijos‐Jaramillo:** conceptualization (lead), data curation (supporting), formal analysis (equal), funding acquisition (lead), investigation (equal), methodology (supporting), project administration (lead), writing – original draft (lead), writing – review and editing (lead).

## Funding

This work was supported by the Universidad de Las Américas Ecuador, PRG.BIO.23.14.01.

## Conflicts of Interest

The authors declare no conflicts of interest.

## Supporting information


**Table S1:** List of articles reporting interkingdom HGT candidates analyzed in this study.
**Table S2:** AVP settings applied to each candidate reanalyzed in this study.
**Table S3:** Assembly statistics associated with each genome/candidate included in this study.
**Table S4:** Classification of phylogenetic patterns and associated statistics for each HGT candidate.
**Table S5:** Interkingdom HGT candidates classified according to relevant Plantae lineages, using the nomenclature established in Table S3.

## Data Availability

The data that support the findings of this study are openly available in the Zenodo repository at https://zenodo.org/records/17552462.
